# The Inhibition Action of Some Brij-Type Nonionic Surfactants on the Corrosion of OLC 45 in Various Aggressive Environments

**DOI:** 10.3390/ma17061378

**Published:** 2024-03-17

**Authors:** Florina Branzoi, Adriana Băran, Marius Alexandru Mihai, Mohamed Yassine Zaki

**Affiliations:** 1 Institute of Physical Chemistry “Ilie Murgulescu”, 202 Splaiul Independenţei, 060021 Bucharest, Romania; adibaran@gmail.com (A.B.);; 2 National Institute of Materials Physics, 405A, Atomistilor Street, 077125 Magurele, Romania; zakimoham@yahoo.com

**Keywords:** corrosion inhibitor, surfactant, electrochemical techniques, OLC 45, SEM, FT-IR

## Abstract

The corrosion protection property of three Brij-type surfactants, namely, Brij 35, Brij 56 and Brij 58P, was considered on OLC 45 carbon steel in a 0.5 M H_2_SO_4_ medium. The efficacy for these organic compounds was examined using potentiodynamic polarization and electrochemical impedance spectroscopy (EIS) methods, scanning electron microscopy (SEM) procedures, and Fourier transform infrared (FT-IR) spectroscopy. We hypothesized that these surfactants hinder the corrosion for OLC 45 samples through a protecting mechanism owing to the adsorption of organic molecules that form an inhibitive film or through the formation of complex oxides. These surfactants exhibited an appreciable protective effect against OLC 45 corrosion, operating as mixed inhibitors, as could be demonstrated by their influence on the electrochemical characteristics of the metallic substrates. The adsorption of surfactants over the substrates zone conformed to the representation of the Langmuir isotherm. The effect of temperature on the electrochemical comportment of the OLC 45 specimens in H_2_SO_4_ without and with Brij at 800 ppm was examined in the temperature interval of 293 to 333 K. The negative estimate of thermodynamic attributed as Gibbs free energy of adsorption presented the spontaneity of the adsorption activity. The investigation with FT-IR and SEM established the adsorption of Brij and the constitution of the corrosive components on the OLC 45 surface. Electrochemical determinations of these surfactants indicated its anticorrosion inhibition performance and the highest inhibition of 96% was reached when the Brij 35 concentration was at 800 or 1000 ppm, while for Brij 56 and Brij 58P, the highest inhibition was obtained when their concentrations were 500, 800, or 1000 ppm.

## 1. Introduction

The corrosion of metals presents a considerable economic and manufacturing preoccupation. Metallic materials have a huge degree of significance for mankind, and have played a decisive role in a diversity of applications due to their flexible attributes and low cost. Metals and their alloys represent a wide area utilized in various technological practices, such as chemical procedures, petroleum manufacturing and purification, industrial machinery, marine processes and automotive industry, and this has enhanced the exploration of corrosion inhibition in several aggressive media [1,2,3,4,5,6,7]. In many industrial practices, the substrates of the materials utilized are exposed to extremely corrosive acid, alkali, and saline environments, which produce (provoke) substantial destruction through corrosion. Since corrosion is the considerable agent in the deterioration of manufacturing constituents, many experiments were conducted to discover mechanisms to reduce corrosion and wear expenses. Protection against the corrosion of these metallic materials can be accomplished using several techniques, among which the treatment of corrosive environments through the application of inhibitors is a significant procedure [8,9,10,11,12,13,14,15]. Numerous investigations performed for the defense of metals used in technological domains have divulged that it is a very efficacious and simple application to utilize organic inhibitors to impede the deterioration of metals in aggressive environments [16,17,18,19,20,21,22,23,24,25].

Organic corrosion inhibitors (amines, amides, quaternary salts, pyridines, pyrazoles, imidazole, benzotriazoles, natural extracts, biomolecules, drugs, and ionic liquids) constitute one of the largest corrosion-examining processes that can greatly diminish the corrosion speed of metals in corrosive environments, owing to their chemical characteristics occurring from their heteroatoms, such as N, O, S, and P atoms, and their ability to coordinate efficaciously with the metal substrate through their lone pairs of electrons and the vacant d-orbital of the iron complex, which places them as a powerful procedure for diminishing the corrosive action on steel in aggressive solutions [26,27,28,29,30,31,32]. The organic substances (inhibitors) proceed through adsorption on the metal substrate and this adsorption can be favored by electrostatic attractiveness through charge dividing or chemical adsorption through back donation of the lone-pair electrons. The adsorption mechanism can be appreciated by explaining some kinetic and thermodynamic characteristics [33,34,35,36,37,38].

The efficacy of these inhibitors can be evidenced by the area covered, which is influenced by the molecule’s geometrical form, electron-donating/retro (back)-donation functional groups, steric elements, aromatic character, and planarity of construction, etc. In recent years, the progress of new friendly organic substances (surfactants, plants extract, polymers) with anticorrosion properties that have ecological, biodegradable, cheap, and healthy characteristics for active materials represents a large domain of exploration in the area of anticorrosion protection [39,40,41,42,43,44]. Surfactants are organic inhibitor molecules which contain hydrocarbonate chains of diversified lengths and these variations of the attached alkyl chains can determine a notable impact on the hydrophobicity of a corrosion inhibitor [45,46,47,48]. The extent of the alkyl chains and the existence of π-bonds, including heteroatoms, execute a huge effect on the adsorption capacity. While the large alkyl chains can lead to increased hydrophobicity, they can also have an agglomerating effect, which can induce problems for inhibitor adsorption. Several studies revealed that Brij surfactants are suitable for a large range of applications including environment depollution [49,50,51,52,53], protein stabilizers, [54,55] and, more importantly, drug delivery systems (due to their biocompatibility) [56,57,58,59]. Since these expire and become unusable, their further processing can provide not only economically advantageous solutions for various applications, but also a way to protect the environment. Therefore, a great way to recycle Brij-containing expired products seems to be their use as corrosion inhibitors, but, at least to our knowledge, literature regarding their corrosion inhibition features is extremely scarce [60,61,62]. Consequently, in this work, we explore and compare the inhibitive properties of Brij 35, Brij 56, and Brij 58P to the corrosion of OLC 45 steel substrates, as these surfactants are widely used in the aforementioned applications. These Brij surfactants are plant-based fatty polyoxyethylene ethers derived from lauryl and cetyl alcohols. Brij surfactants are stable to acids and alkalis beyond the pH domain that ester type emulsifiers can resist. Hence, Brij is helpful for emulsifying fats and oils in highly acidic or alkaline media. Its physical properties can be presented as follows: all are waxy solids; Brij 35: CMC 0.08 mM, HLB 16.9, mp = 43 °C, ρ =1.05 g/cm^3^; Brij 56: CMC 0.035 mM, HLB 12.9, mp = 31 °C, ρ = 0.98 g/cm^3^; and Brij 58P: CMC 0.075 mM, HLB 15.7, mp = 38 °C, ρ =1.01 g/cm^3^. The aim of this study was to find new “environmentally friendly” organic compounds suitable for the corrosion protection of base metals. Another area of interest was the optimization of the best anticorrosion characteristics for some materials in aggressive environments.

The reason for this research was to explore the corrosion protection performances of three nonionic Brij-type surfactants (Brij 35, Brij 56, Brij 58P) as corrosion inhibitors for the protection of OLC 45 in 0.5 M H_2_SO_4_ environments. The inhibition capacity of these Brij surfactants was appraised with electrochemical impedance spectroscopy, polarization curves, spectroscopy, scanning electron microscopy, and Fourier transform infrared procedures. Moreover, the effect of the Brij concentration, immersion period, and temperature on the corrosion protection was investigated.

## 2. Experimental

### 2.1. Materials

**Scheme 1:**
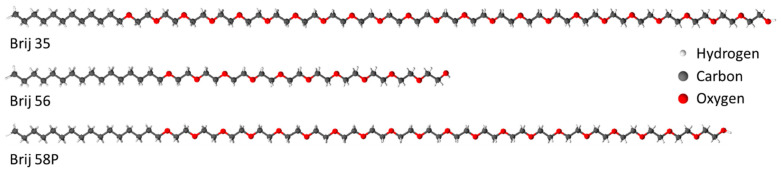
The chemical structures of Brij35-CH_3_(CH_2_)_11_(OCH_2_CH_2_)_23_OH, Brij 56-CH_3_(CH_2_)_15_(OCH_2_CH_2_)_10_OH and Brij 58P-CH_3_(CH_2_)_15_(OCH_2_CH_2_)_20_OH.

In this work, the organic compounds examined included three surfactants: Brij 35 (polyethyleneglycol (23) lauryl ether, C_12_H_26_ (CH_2_CH_2_O)_23_OH), Brij 56 (polyethyleneglycol (10) monocethyl ether, C_16_H_33_(CH_2_CH_2_O)_10_OH), and Brij 58P (polyethyleneglycol (20) hexadecyl ether C_16_H_33_ (CH_2_CH_2_O)_20_OH) (Scheme 1) were produced from Sigma-Aldrich of pure quality (>98%). The 0.5 M H_2_SO_4_ aggressive environment was made by diluting 97% H_2_SO_4_ (AG, Merck, Darmstadt, Germany) with bidistilled water. The substances were of reagent grade and utilized as purchased without supplementary purification. An ALJ 120-4 analytical balance (0.1 mg resolution, 0.2 mg reproducibility, and ±0.2 mg linearity, KERN, Eschenlohe, Germany) was employed for the solutions preparations. Corrosion determinations were made on OLC 45 substrate with the following composition: C% 0.48, Si 0.03%, Mn 0.79%, Fe% 98.32, P% 0.02, S% 0.025, Al% 0.027, Ni% 0.05, Cr% 0.06, Cu% 0.18, Sn% 0.012, and As% 0.006. The OLC 45 carbon steel sample was cylindrical in shape and had an area of 0.5 cm^2^. This form was preferable as it provided a substantial and borderless surface. The OLC 45 sample was mechanically abraded with sandpapers of various sizes (600–4000 grit) to a mirror finish (shine). Next, the OLC 45 samples were cleaned in benzene until all the greasy residue (fatty remainders) was removed; after that, the working specimen was rinsed in double-distilled water, dried at ambient temperature, and positioned in the work cell. All experiments were performed at 25 °C in atmospheric oxygen with non-agitation.

### 2.2. Methods and Instruments

Corrosion determinations were performed with and without some surfactant dosages. The electrochemical experiments were effectuated on an electrochemical cell with three typical electrodes: a platinum plate auxiliary electrode, a saturated calomel reference electrode, and OLC 45 with an area of 0.5 cm^2^ as the working electrode. The work cell was connected to a VoltaLab 40 model automatic potentiostat/galvanostat linked to a computer working VoltaMaster 7.09 software. The electrochemical performance of an OLC 45 specimen in 0.5 M H_2_SO_4_ without and with three Brij surfactants was examined by recording anodic and cathodic potentiodynamic polarization procedures and electrochemical impedance spectroscopy determinations. The protective activity was examined using polarization curves made using potentiodynamic measurements and evaluation of the electrochemical characteristics for OLC 45 samples with and without certain doses of surfactants. Exploration of Tafel polarization curves was performed through potential translation from cathodic to anodic potential for OCP (±250 mV OCP) at a scan rating of 2 mV/s. The Tafel branches of the anodic and cathodic plots were extrapolated at corrosion potential (E_corr_), and the corrosion current density (i_corr_) and anodic and cathodic Tafel slopes (b_a_, b_c_) were acquired. All potentials were registered with respect to SCE. Electrochemical impedance spectroscopy determinations were attained for OCP over the frequency interval of 100,000 Hz to 0.04 Hz with an AC wave of ±10 mV (peak-to-peak), and the impedance assays were realized at a rate of 10 points per decade with varying frequency. The measurements were rehearsed for every sample to achieve a good concordance of the result. (Each determination was performed three times to account for reproducibility.) A VoltaLab-PGZ402 (Radiometer Analytical, France) potentiostat/galvanostat system was utilized in all electrochemical determinations.

**Scheme 2:**
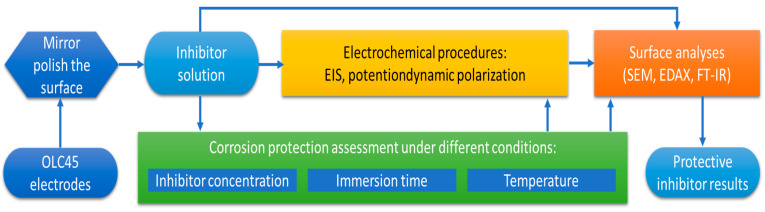
Examination and characterization of Brij surfactants on OLC 45 substrate in corrosive medium.

The protective layer of the inhibited sample was measured using a Bruker Optics Tensor 37 FT-IR spectrometer (with ATR), Ettlingen, Germany in the spectral interval 4000–650 cm^−1^, to a resolution of 4 cm^−1^. The morphology of the defensive film of these Brij-type surfactants on OLC 45 substrate was explored using scanning electron microscopy and metallographic micrographs using a Hund H660 microscope (Wetzlar, Germany). Substrate morphology examinations were executed using SEM in a dual-beam FEI Quanta 3D FEG model (Brno, Czech Republic) with an energy-dispersive X-ray (EDX) spectrometer working in high-vacuum mode with an accelerating voltage of 2 to 30 kV. Bare specimen preparation involved holding the specimen on double-sided carbon tape without a coating. Scheme 2 is described below for the investigation and characterization of Brij surfactants as a protective inhibitor on the substrate of OLC 45 specimen in aggressive environment.

## 3. Results and Discussion

### 3.1. Electrochemical Studies

#### 3.1.1. Potentiodynamic Polarization Procedures

**Figure 1:**
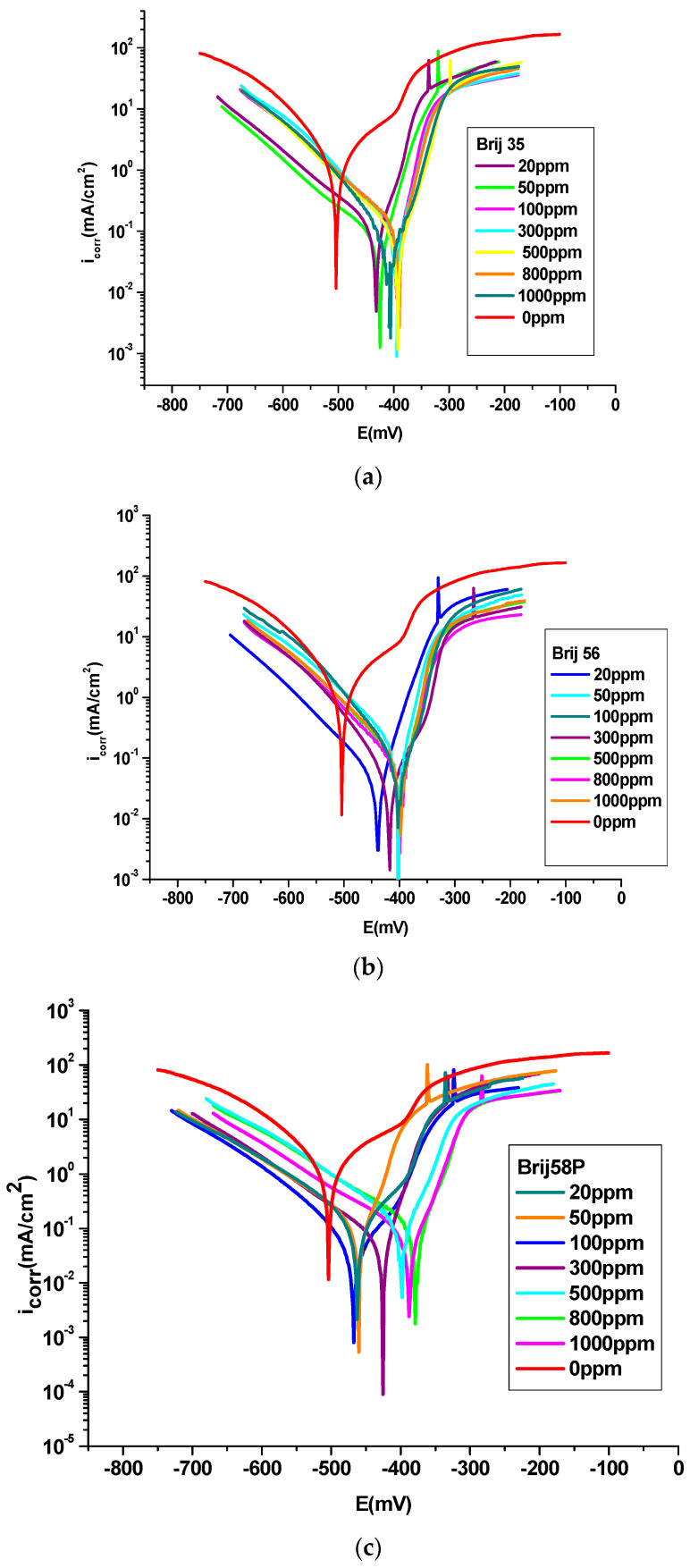
Polarization curves of OLC 45 sample in 0.5 M H_2_SO_4_ at different concentrations of (**a**) Brij 35, (**b**) Brij 56, and (**c**) Brij 58P at 25 °C.

**Figure 2:**
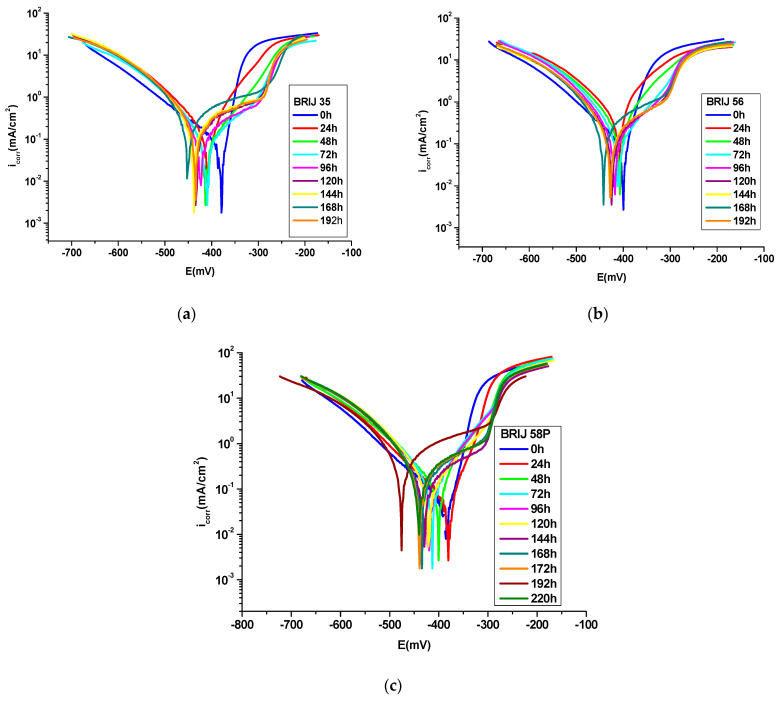
Polarization curves for OLC 45 sample in 0.5 M H_2_SO_4_ at 800 ppm concentration of (**a**) Brij 35, (**b**) Brij 56, and (**c**) Brij 58P with different immersion times (at 25 °C).

In this exploration, one of the greatest protection procedures against the corrosion of OLC 45 substrates in aggressive environments, the use of the organic compounds, was inspected in terms of corrosion in the anodic or the cathodic process, or both. The anticorrosive properties of these Brij-type surfactants on the OLC 45 sample were estimated in 0.5 M H_2_SO_4_ using a potentiodynamic polarization practice and EIS. The polarization curves of uninhibited and inhibited OLC 45 samples in 0.5 M H_2_SO_4_ solution are shown in Figure 1 and Figure 2. The surfaces inhibited by these Brij surfactants revealed a considerable decrease in the anodic and cathodic current, which led to the diminution in the cathodic and anodic mechanisms. It was determined that the anodic and cathodic bias curves distinguished a reduced current density of the Brij surfactants than those considered in the uninhibited samples. This behaviour disclosed that all the Brij-type surfactants used had an important action in the anodic and cathodic processes for the electrochemical procedure. From Figure 1, it can be seen that the anodic metal dissolution and cathodic hydrogen release procedures were hindered through the introduction of these chemical compounds into the aggressive solution. The attendance of surfactants Brij 35, Brij 56, and Brij 58P as corrosion protectors also changed the corrosion potential (E_corr_) to be more positive compared to the uninhibited sample, revealing that the surfactants possess a greater influence in the anodic process than the cathodic one.

Electrochemical characteristics, such as the corrosion current density (i_corr_), cathodic and anodic Tafel slopes, corrosion potential (E_corr_), and protection efficiency (E_%_), are introduced in Table 1, Table 2 and Table 3. It can be seen in Figure 1 and Table 1, Table 2 and Table 3 that the addition of Brij35, Brij 56, and Brij 58P changed the corrosion potential, displacing the E_corr_ to become more positive and remarkably diminishing the inhibited anodic Tafel slopes (b_a_); the existence of surfactants manifested an impact on the anodic dissolution action of the metal [17,18,19,20,21,22,23,24]. This case may be assigned to the adsorption of the SO_4_^2-^ ions and/or inhibitory molecules on the anodic active of the OLC 45 zone and the impedance of the obstruction of the anodic metal dissolution action. Experimental data designated that the corrosion current was considerably diminished with the concentration of nonionic surfactants Brij 35, Brij 56, and Brij 58P, and the inhibition efficacy increased. The experiments divulge that the constitution of the protective layer was adsorbed on the OLC 45 substrate, obstructing the available active seats (centers).

Considering the comparison of the inhibition efficiency and the corrosion speed (Rmpy, in mil/year; P, in mm/year and Kg, in gm^−2^h^−1^) for all surfactants, under the same circumstances it is evident that Brij 35, Brij 56, and Brij 58P display efficacious corrosion defense for the OLC 45 substrate in 0.5 M H_2_SO_4_. The surfactants show a greater protective effect against metal corrosion since they have long-chain carbon bonds and certain adsorption places (oxygen atoms) which cause considerable adsorption onto the OLC 45 substrate, and they are influenced by the type of donor/acceptor action [31,32,33,34,35,47]. The protective activity was evidenced by the adsorption of the inhibitory molecules on the active places and/or the formation of corrosion products on the surface of the OLC 45 sample. It can also be observed from Figure 1 and Table 1, Table 2 and Table 3 that a higher protection efficacy was obtained for Brij 35/OLC 45 at concentrations of 1000 ppm and 800 ppm; for Brij 58P/OLC 45 at concentrations of 800 ppm, 1000 ppm, and 500 ppm; and for Brij 56/OLC 45 at concentrations of 800 ppm and 1000 ppm. These surfactants showed the best inhibition properties for the OLC 45 sample in an acidic environment (0.5 M H_2_SO_4_), when the corrosion current was diminished and the protection performance increased with a rising surfactant concentration. The corrosion protection efficiency of Brij 35 was very close to that of Brij 58P, and both were superior to Brij 56. On closer inspection, it appears that Brij 35 had the highest performance at low concentrations, whereas Brij 58P was superior at higher concentrations (see Table 1, Table 2 and Table 3). Most probably, the main reason for this behavior comes from the size of the polar chain of the surfactant, as at a higher number of oxyethylene groups, many active centers for the adsorption processes of these substances should result in a stronger interaction between the inhibitor and the substrate (see Langmuir isotherm). This is consistent with the electrochemical measurements (Table 1, Table 2 and Table 3) which revealed that Brij 35 (23 oxyethylene groups) had the highest affinity for the OLC 45 substrate, followed by Brij 58P (20 oxyethylene groups), and ultimately by Brij 56 (10 oxyethylene groups). Therefore, it appears that at low concentrations the adsorption effect dominates, rendering Brij 35 the most efficient inhibitor. As the concentration increases and the surface coverage of the of the substrate becomes saturated, adsorption can no longer play an important role and the repulsive effect of the non-polar chain becomes prevalent, turning Brij 58P into the superior corrosion inhibitor (length of alkyl chain of B58P = 16 vs. length of alkyl chain of B35 = 12). Unsurprisingly, Brij 56 was the least effective corrosion inhibitor among the ones analyzed, since it neither the highest number of oxyethylene groups nor a longer alkyl chain (length of C alkyl B56 = 16). Furthermore, the protective performance increased with the hydrophobic molecule chain length, and, when the surfactant dosage was at a concentration higher than the critical micelle concentration (CMC) the inhibitory activity of these Brij-type substances rises rapidly. The appearance of the hydrocarbonate chains of the Brij that “competitively adsorb” onto the OLC 45 substrate obstructs the active centers and, as an effect, the SO_4_^2−^ (corrosive element) is impeded from offending the OLC 45 substrate, ensuring protection.

#### 3.1.2. Influence of Immersion Time

**Figure 3:**
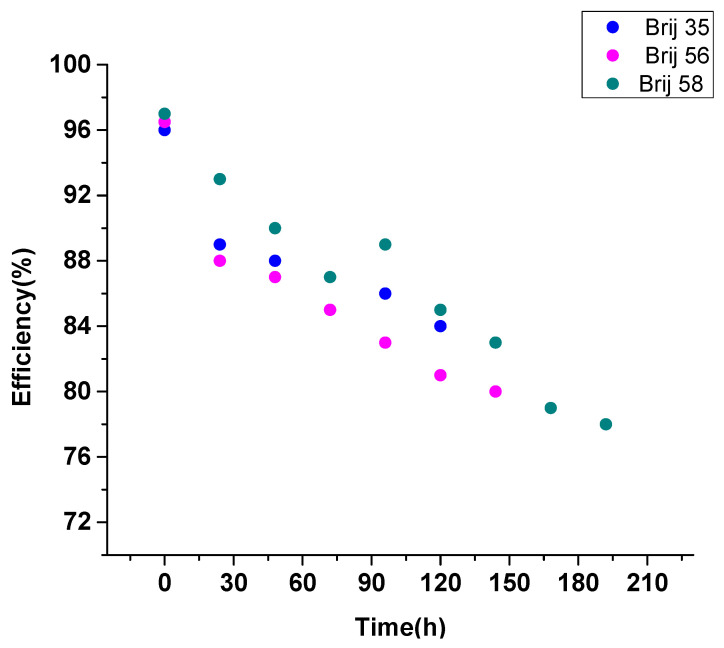
Presentation of the influence of protection efficiency to immersion period for OLC 45 in 0.5 M H_2_SO_4_ with Brij 35, Brij 56, and Brij 58P surfactants.

Using potentiodynamic polarization, the result of a period of increased immersion (0–192 h) for the corrosion inhibition of these Brij surfactants at an 800 ppm concentration for the corrosion of OLC 45 in H_2_SO_4_ at 25 °C was studied. The protective performance diminished slowly and a slight increase in the corrosion speed with rising immersion period can be observed in Figure 2. This was due to the deterioration of the inhibitory layer with the rising immersion period, as a consequence of the transformation in the active zone. This may have been due to certain defects existing on the inhibitory film that afforded the entry of the aggressive element (SO_4_^2−^) at the OLC 45/surfactant interface. Additionally, the constitution of hemimicelles aggregates by the first established surfactant molecules reduces the substrate covered by the organic substance. The outcome of the inhibition efficiency of the three Brij surfactants after immersion is depicted in Figure 3. It is clear that at an immersion time of 120 h, the efficiency of the Brij surfactants was still 80%, which shows that the nonionic surfactants are long-lasting time-protective inhibitors for OLC 45 in a 0.5 M H_2_SO_4_ environment. Consequently, the inhibited substrate can successfully protect the OLC 45 from corrosion for a long time.

#### 3.1.3. Electrochemical Impedance Spectroscopy (EIS)

**Figure 4:**
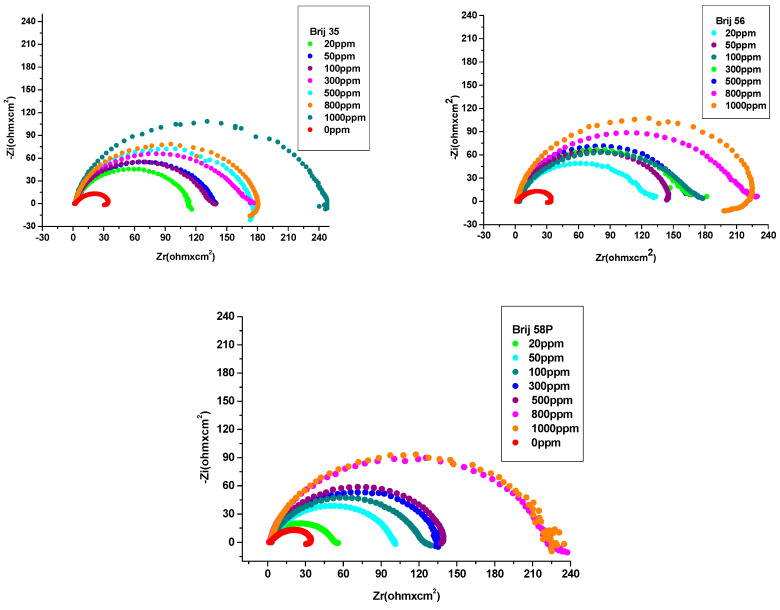
Nyquist diagrams for OLC 45 in 0.5 M H_2_SO_4_ medium with some concentrations of Brij 35, Brij 56, and Brij 58P at 25 °C.

The protective activity of three surfactants onto the OLC 45 sample in H_2_SO_4_ environment was explored using electrochemical impedance spectroscopy (EIS). The EIS determinations detailed the protection effect of Brij surfactants as an anticorrosive protector of the OLC 45 specimen in aggressive solutions. EIS data provide an evaluation of the inhibitory property of surfactants through the corrosion protective film. Nyquist diagrams for the OLC 45 obtained at the interface in the presence and absence of certain amounts of surfactant are displayed in Figure 4. 

It can be seen in Figure 4 that the Nyquist graphs on the OLC 45 specimen indicate a small capacitive loop, showing that the “charge transfer” activity was dominated by the corrosion operation (action). The Nyquist plots for OLC 45 with surfactants denote a capacitive loop that is representative of a charge transfer procedure. Mainly, the capacitive loop as established in the Nyquist plots presumes a single constant most probably corresponding to the charge transfer reaction of the inhibitory surfactants on the OLC 45 substrate. Therefore, the sizes of the capacitance loops of the inhibited substrate were higher than those of the uninhibited OLC 45 electrode, and the measurements of these loops increased with the surfactant dose, suggesting that these Brij surfactants provided greater protection results on the OLC 45 specimen in H_2_SO_4_. It is noticeable from the Nyquist plots that the impedance reaction of the OLC 45 specimen was significantly changed through the addition of the Brij surfactants, which indicates that the defensive film was confirmed through the presence of Brij 35, Brij 56, and Brij 58P surfactants. From Figure 4, it can be seen that the impedance diagrams are not complete semicircles and this fact is attributed to the frequency dispersion, mostly due to roughness and inhomogeneities in the OLC 45 area [23,31,32,33,34,35,36,37,47]. Figure 4 reveals that the capacitance loop diameters at 1000 ppm and 800 ppm for Brij35, at 800 ppm and1000 ppm for Brij 56, and at 800 ppm and 1000 ppm for Brij 58P were larger than those in the absence of surfactants, suggesting that Brij provides a better protection effect of the sample in H_2_SO_4_.

**Figure 5:**
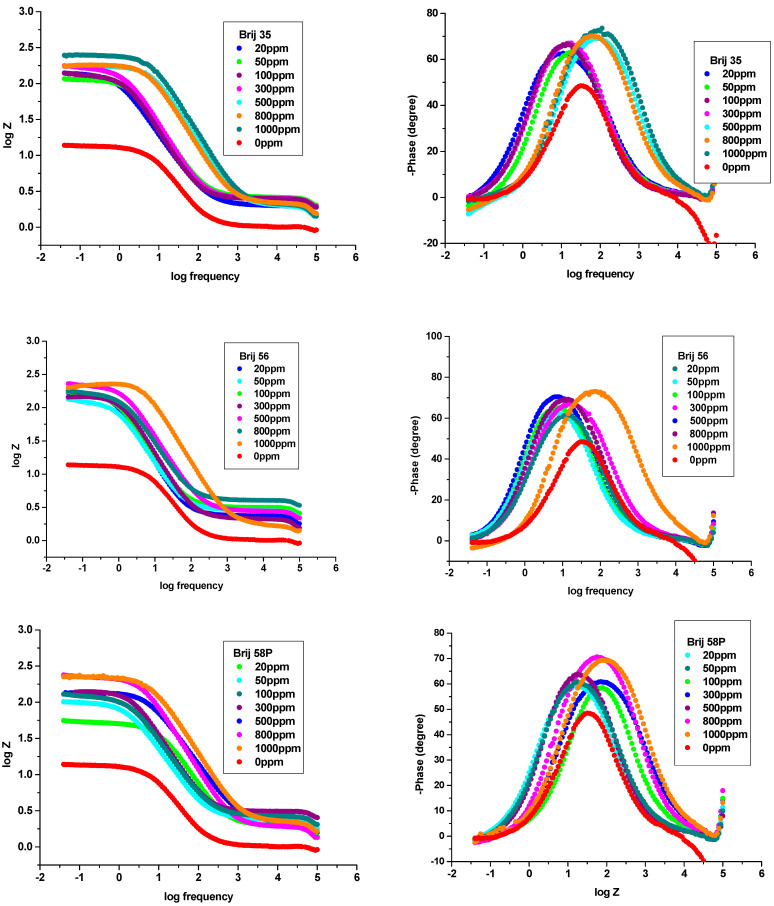
Bode graphs of OLC 45 in 0.5 M H_2_SO_4_ at some concentrations of Brij35, Brij 56, and Brij 58P at 25 °C.

**Figure 6:**
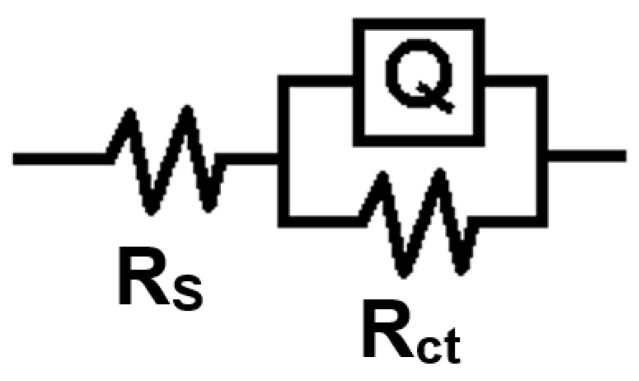
Equivalent circuit.

The Bode plots of the OLC 45 sample uninhibited and inhibited using Brij (Figure 5) indicate that the impedance modulus, at low frequencies, increased with an increasing concentration of these Brij-type inhibitors, displaying that the adsorption of Brij surfactant molecules raises the corrosion defense of the OLC 45 substrate in an acidic electrolyte. There is only one time constant in the Bode plots, exemplifying that the permeated solution did not attain the OLC 45 substrate and no corrosion had prevailed on the substrates [11,12,23]. In Figure 5, it is evident that the OLC 45 specimen only shows one time constant at a phase angle of 47° at medium and low frequencies, indicating an inductive behaviour through a poor diffusion tendency. The Bode plots from Figure 5 display that the attendance of nonionic surfactants (Brij 35, Brij 56, Brij 58P) on the phase angle for the logarithm of frequency manifested a well-defined maximum at a phase angle of 70–80°, which correlates with a relaxation time constant that assumes a large capacitive behaviour. In the Bode plots, the phase angle significantly increased with the Brij surfactants due to obtaining the defense film over the OLC 45 substrate. As a consequence, under these circumstances, the inhibited substrates possessed a large capacitive comportment according to the Nyquist determinations and the results of the potentiodynamic polarization practice. The increase in Z_mod_ designates a superior inhibitive capability and it is also obvious that Z_mod_ increases when the concentration of all elaborated surfactants rises. A higher Z_mod_ demonstrates a higher protection performance. The equivalent electrical circuit of R(QR) displayed in Figure 6 was utilized to fit the EIS spectra, since only one time constant was evident in the Bode diagrams. The assessment of the impedance data was established using suitable results with the corresponding equivalent circuit and several parameters such as the solution resistance (Rs), the charge transfer resistance (Rct), and the capacitance of the double layer (Cdl), which have been presented in Table 4, Table 5 and Table 6. In this study, an example frequency domain equivalent circuit developed to match and account for the obtained EIS data was suggested. In this occurrence, Q (CPE), the constant phase element, was revealed to simulate the non-ideal capacitance behaviour. The CPE was applied to establish the deformity of the capacitance loop by assigning the heterogeneity of the area to substrate ruggedness and impurity. The impedance PE can be defined as Z_CPE_ = Y_0_^−1^ (jω)^−n^, where Z_CPE_ is the impedance of the CPE, ω is the “angular frequency”, “j” is the “imaginary number” (j^2^ = −1), Y_0_ is the corresponding amplitude at a capacitance, and “n” is the “phase shift”. The “n” rating describes the inhomogeneity state of the substrate area. A superior rating of “n” is related to a lower degree of rugosity of the substrate i.n., and the non-homogeneity of the substrate is low. The CPE is the resistance when n = 0, (Y_0_ = R), the capacitance when n = 1 (Y_0_ = C), and the inductance when n = −1 (Y_0_ = 1/L), or the Warburg impedance when n = 0.5 (Y_0_ = W), according to the appreciation of “n” [11,12,33,34,35,36,37].

The EIS tests showed that the charge transfer resistance, Rct, increased and the double layer capacitance, Cdl, diminished due to the inhibition of OLC 45 substrates with Brij surfactants. The results establish that with the increase in Rct using the dosage of Brij surfactants, the protective effect increased considerably, which proves that surfactants have a considerable anticorrosion impact for the OLC 45. The diminution of Cdl can be realized by lessening the local dielectric constant and/or increasing the thickness of the electrical double layer, due to the fact that surfactant operates through adsorption on the sample/electrolyte interface. Brij surfactants adsorbed to the surface of OLC 45 samples and established an inhibitory layer on the OLC 45 surface. By increasing the value of Rct, the inhibition efficiency also improved. The Nyquist and Bode plots denote that corrosion activity was obstructed by Brij 35, Brij 56, and Brij 58P surfactants, and that this action was attained as a “diffusion barrier” and through a charge transfer activity.

### 3.2. The Influence of Temperature

**Figure 7:**
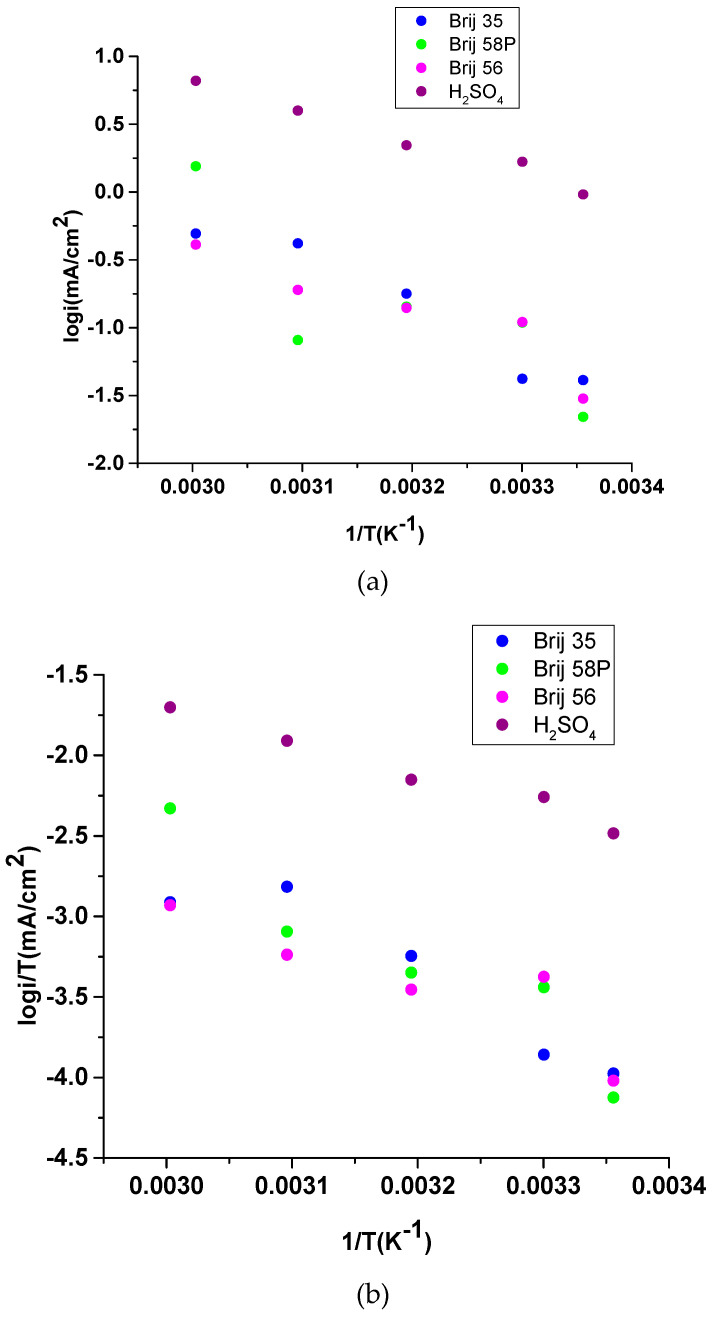
Arrhenius graph for OLC 45 in 0.5 M H_2_SO_4_ in absence and presence of Brij 35, Brij 56, and Brij 58P at different temperatures.

The impact of temperature on the protection performance of Brij 35, Brij 56, and Brij 58P surfactants at a dosage of 800 ppm for OLC 45 in 0.5 M H_2_SO_4_ at temperatures of 298 K, 303 K, 313 K, 323 K, and 333 K was examined with a potentiodynamic polarization practice. It can be seen that the corrosion speed grew by raising the temperature in the inhibited and non-inhibited electrolyte. The inhibitory activity of the Brij surfactants decreased with increasing temperatures, while the protective performance of these surfactants for OLC 45 in a corrosive medium was affected through the surfactant adsorption; however, superior temperatures caused the desorption of the Brij 35, Brij 56, and Brij 58P inhibitors from the OLC 45 surface. The modification in corrosion rate as a function of temperature can be expressed using the Arrhenius relationship and a transition formula [14,15,23,47]:

$$i_{corr}=A\exp\left(\frac{-E_{a}}{RT}\right)$$

$$i_{corr}=\frac{RT}{Nh}\exp\left(\frac{\Delta S_{a}^{*}}{R}\right)\exp\left(\frac{\Delta H_{a}^{*}}{RT}\right)$$

where i*_corr_* is the rate of procedure, A is a pre-exponential element, E_a_ is the “apparent activation energy” of the OLC 45 dissolution mechanism, T is the absolute temperature, R is the universal gas constant, $\Delta H_{a}^{*}$ is the “apparent enthalpy of activation”, $\Delta S_{a}^{*}$ is the “apparent entropy of activation”, h is the Planck’s constant, and N is the Avogadro number. Figure 7a represents the Arrhenius graph of the corrosion rate for 1/T for the OLC 45 specimen in 0.5 M H_2_SO_4_ with and without the three Brij surfactants. Estimates of E_a_ without and with these Brij surfactants were acquired by plotting the corrosion rate for 1/T, wherein straight lines were drawn (Figure 7a), and using the slope of these lines to determine the activation energy (Table 7). Figure 7b illustrates a plot of the logarithmic corrosion rate/T for 1/T. The lines were made with a slope of (−ΔH*/R) and an intercept of (ln(R/Nh) (ΔS*/R), where from ΔH* and ΔS* a worth was estimated (Table 7). Inspecting Table 7 and Figure 7 reveals that the E_a_ ratings were increased with Brij compared to those which lacked the surfactants, so that the E_a_ rating can represent the impact of temperature for protective activity.

The greater E_a_ values in the corrosion process with these Brij surfactants highlights the main protective action of these surfactants. With the adsorption data, it can be considered we found the chemisorption and physisorption of surfactants on the OLC 45 substrate. The energy barricade for the corrosion mechanism increased and the surfactant molecules were adsorbed onto the OLC 45 substrate, which reduced the interaction between the aggressive environment and the OLC 45 substrate.

The superior value for E_a_ in the inhibited environment can be explained by the increases in the thickness for the double layer, which realized the activation energy for the corrosion activity. It demonstrates that the diminishment in effectiveness with rising temperatures can be ascribed to the increased desorption of surfactant molecules from the substrate of the OLC 45 sample. The positive enthalpy value suggests that the endothermic aspect of the metal dissolution mechanism and the dissolution procedure of OLC 45 was diminished by the surfactant and that the dissolution of this sample is complex. The greater and negative value of ΔS° with the surfactant and in its absence divulges that the activated complex in the rate-setting step determines the association rather than the dissociation step, implying a diminution in the disorder if the transition from reactant to complex is operated by acquiring a constant adsorption film of surfactant molecules on the surface of the OLC 45 substrate.

### 3.3. Adsorption Isotherm

The adsorption isotherm supplies relevant insight into the interaction for the organic compound and the sample surface. Furthermore, the higher effectiveness of the surfactant was a consequence of the adsorption action. It is fully assumed that the adsorption of the surfactant onto the OLC 45 substrate is the important activity in the defense mechanism. To estimate the effect of the surfactant concentration for corrosion protection, it was necessary to fit the rate results to the adsorption equilibrium relationship, in the form of a Langmuir isotherm type. The excellent connection between the covered substrate and expression of the isotherm was obtained by applying the Langmuir adsorption isotherm.

The Langmuir isotherm is significant to consider the adsorption mechanism based on the following relationship: θ/(1 − θ) = KC, wherein C is the concentration of surfactant, θ is the degree of coverage on the sample substrate with the surfactant, and K is the adsorption equilibrium constant. θ is acquired as follows: θ = (i_corr_ – i_inh_)/i_corr_, where i_corr_ and i_inh_ are the corrosion current in 0.5 M H_2_SO_4_ without and with the surfactant [23,33,34,35,36,37,38]. All the correlation coefficients (R^2^) being greater than 0.99 (Brij 35 R^2^ = 0.9998; Brij 56 R^2^ = 0.9998; and Brij 58P R^2^ = 0.9999) revealed that the defense was ascribed for adsorption of these Brij surfactants to the sample substrate. The practicability of the Langmuir behaviour is often determined on the grounds that protection involves adsorption. The first action step of the metal substrate corrosion activity in 0.5 M H_2_SO_4_ by surfactants is as follows:

Me + INH↔Me(INH)ads ↔Me^n+^ + ne^−^ + INH. (Me = Fe, INH = Brij 35, Brij 56, Brij 58P)

**Figure 8:**
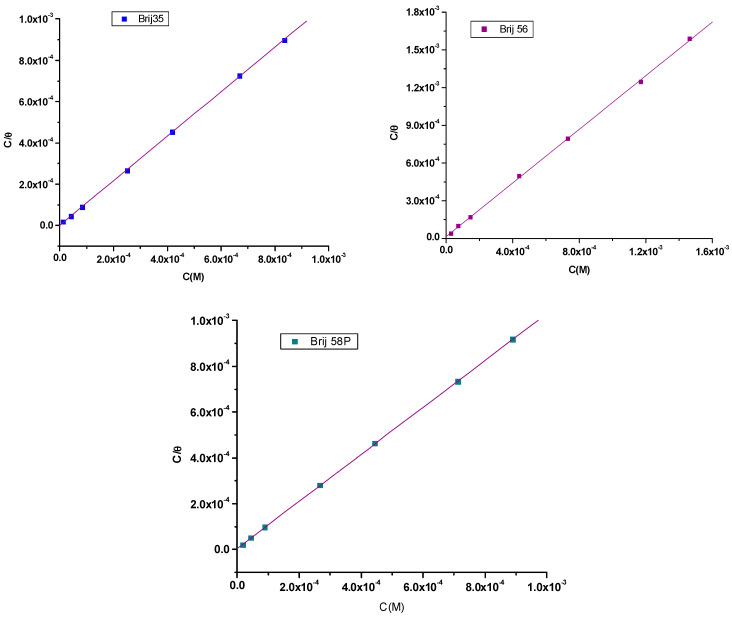
Langmuir plot for Brij 35, Brij 56, and Brij 58P on OLC 45 in 0.5 M H_2_SO_4_.

In the case of an appreciable dose (dosage) of surfactant, a dense and durable film is formed on the OLC 45 substrate sample that decreases the aggressiveness of the OLC 45 specimen. For this exploration, straight lines were acquired where the concentration C_inh_/θ was plotted versus C_inh_ with a slope of unity. The linear relationship suggests that the adsorption of surfactants obeys the Langmuir isotherm (Figure 8). The K_ads_ equilibrium constant with the adsorption process of these Brij surfactants could be established from the reciprocal of the intercept and its evaluation is displayed in Table 8. It is evident that the large values of K_ad_ represent a good adsorption, a superior defense performance of the Brij surfactants on the OLC 45 substrate in 0.5 M H_2_SO_4_, and an intense electrical interaction of the existing double layer and the adsorbed compounds. The adsorption of the Brij surfactant especially amended the corrosion resistance status of a metallic material. The K_ads_ was introduced in the $\Delta G_{ads}^{\circ }$ (standard free energy of adsorption) realized by the following relationship: $\ln K_{ads}=-\left(\Delta G_{ads}^{o}/RT\right)$.

The determined worth of $\Delta G_{ads}^{\circ }$ is negative and establishes that the adsorption of Brij surfactant is a spontaneous procedure, and, in addition, the negative values of $\Delta G_{ads}^{\circ }$, designate the strong interaction of the surfactant molecule on the substrate. The attained worth of approximately −20 KJmol^−1^ or less reveals that the electrostatic interaction in the charged surface of the sample charged most of the electrolyte (physical adsorption), while those of approximately −40 KJmol^−1^ or more implied charge sharing or charge allocation between the substrate OLC 45 and surfactant molecules to constitute a type of coordination bond (chemisorption, Table 8) [33,34,35,36,37,38,39].

#### 3.3.1. Mechanism of Inhibition

**Scheme 3:**
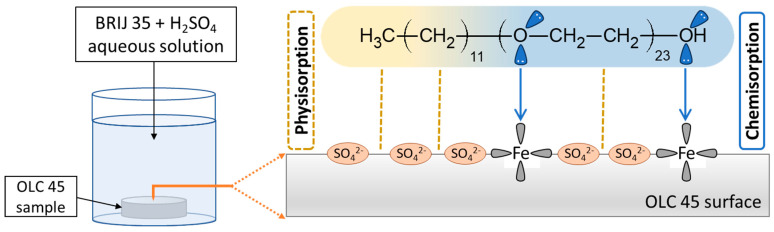
Representation of the adsorption procedure of BRIJ 35 for OLC 45 substrate in H_2_SO_4_ medium.

The inhibition mechanism can be explained by the determinations, and it has been confirmed for all the surfactants considered (Brij 35, Brij 56 and Brij 58P) that they obstructed the corrosion of OLC 45 in 0.5 M H_2_SO_4_ through the adsorption of these surfactants at the substrate/electrolyte interface. Adsorption of these Brij surfactants was achieved using a physical and chemical adsorption procedure. The adsorption activity was controlled by some agents, such as the particularity and charge of the substrate, the chemical constitution and charge of the organic molecules, and the variety of environments. The defense of these surfactants for the metal substrate from corrosion in 0.5 M H_2_SO_4_ was realized through the series of adsorption zones, molecular dimensions, and the mode of interaction used with the metal substrate. It can be emphasized that the large size and high molecular magnitude of a long hydrophobic chain of surfactants can also affect the superior protection performance of the explored Brij. In the case of these Brij surfactants, the existence of functional groups, such as heteroatom O, can be active centers for the adsorption processes of these substances. The O (oxygen) atom has the largest negative charge and it possesses the most considerable ability to settle on the metal area, and is directly adsorbed onto the metal substrate. The protection of these surfactants can be performed by several adsorption modes: through physical adsorption from the negatively charged of the surfactant and/or (SO_4_^2−^), and the positively charged metal substrate; through the chemical interaction on donor–acceptor π-electrons of the O donor atoms of the surfactants and the available d-orbital of Fe substrate atoms; through the large hydrophobic chain of surfactant determined to protect by replacing the water molecule on the substrate OLC 45 with the tendency of these marked alkyl chains to a corrosive environment and to obstruct the diffusion of the corrosive ions -SO_4_^2−^ from the electrolyte [23,33,34,35,36,37,38]. All adsorption categories will diminish the area exhibited to the corrosive environment, so that damage can be stopped. For the protection procedure of Brij 35 for OLC 45 in a H_2_SO_4_ environment, a form of adsorption and inhibition is proposed, as displayed in Scheme 3 (inhibition mechanisms are suitable for Brij 58P and Brij 56).

#### 3.3.2. Comparison of Inhibition Efficiency of Some Nonionic Surfactants with Other Previously Published Corrosion Surfactants

The protective performance of some nonionic surfactants for carbon steel and other substrates in 0.5 M H_2_SO_4_ was comparable (Table 9), and even better than many surfactants. Therefore, surfactants with a higher to critical micelle concentration (CMC) will diffuse out of the bulk water phase and are adsorbed on the interface among carbon steel and the aggressive environment.

### 3.4. Examination of the Surface using FT-IR Spectroscopy

**Figure 9:**
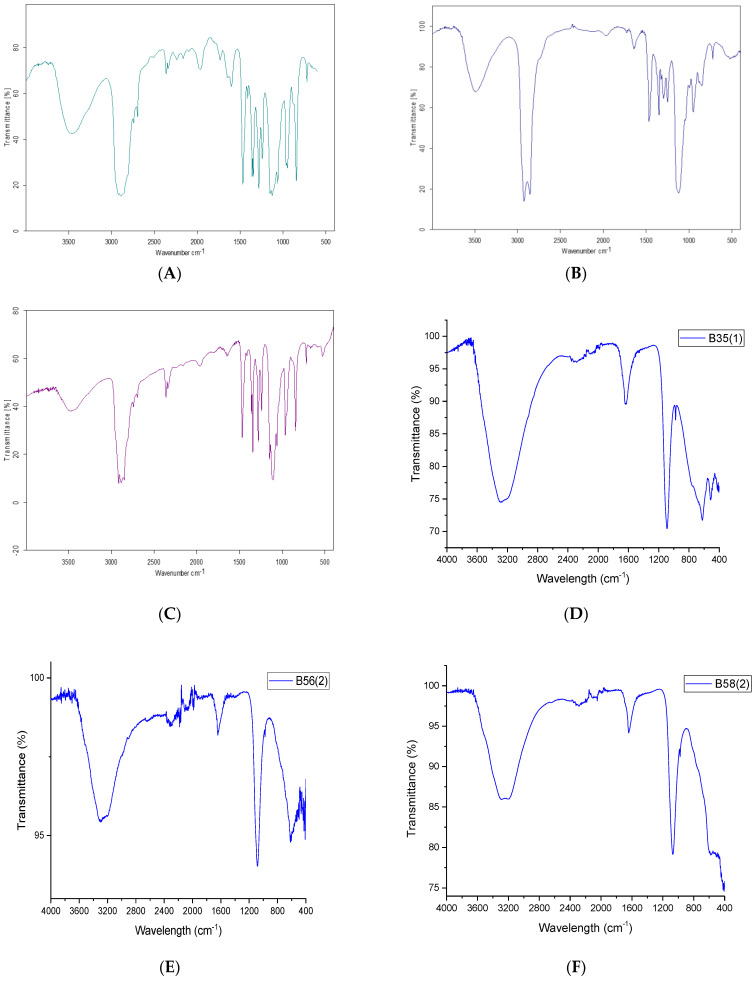
FT-IR transmittance plots of Brij 35 (**A**), Brij 56 (**B**), Brij 58P (**C**), 800 ppm Brij 35 (**D**), 800 ppm Brij 56 (**E**), and 800 ppm Brij 58P (**F**).

In this exploration, an FT-IR procedure was performed to establish the substantial absorption bands noticed for Brij 35, Brij 56, and Brij 58P surfactants adsorbed onto an OLC 45 substrate through immersion in the aggressive environment. To evaluate the inhibitory film acquired on the substrate of the OLC 45 sample using Brij surfactants, and to provide new knowledge about binding on the surface of the OLC 45 specimen, we explored this using FT-IR plots which are depicted in Figure 9. The considerable bands in the transmittance spectra of three surfactants: Brij 35, Brij 56, and Brij 58P pure are depicted in Figure 9A–C.

The characteristic peaks in the FT-IR plots of the three pure Brij surfactants are displayed in Figure 9A–C and the broad absorption bands between 3500 cm^−1^ and 3400 cm^−1^ correspond to an OH group. The peaks around 2900 and 2800 cm^−1^ are allocated to the aliphatic of -CH_3_ and -CH_2_ “symmetric and asymmetric stretching vibration”, and the band at 1500 cm^−1^ is allocated to the C-H stretching vibration (Figure 9A–C). The band from 1200 to 1300 cm^−1^ is assigned to C-H bending and the peak at 1100–800 cm^−1^ is ascribed to a C-O-C vibration in (CH_2_CH_2_O)n (Figure 9A–C). The FT-IR data for the adsorbed defense film procured onto the OLC 45 substrate through immersion in corrosive environments encompassing an optimal concentration of 800 ppm Brij 35, Brij 56, and Brij 58P are presented in Figure 9D–F. The peaks displayed at 3282 cm^−1^, 3299 cm^−1^, and 3301 cm^−1^ correlated to O-H stretching. The indicative peaks at 2989 cm^−1^, 2994 cm^−1^, and 2991 cm^−1^ were designated to the aliphatic symmetric stretching vibration of -CH_3_ and -CH_2_. The “absorption bands” placed at approximately 1200 cm^−1^ were represented in the “stretching vibration” of the C-O (Figure 9D–F). The peaks revealed at approximately 1090 cm^−1^, 1080 cm^−1^, and 1065 cm^−1^ could be attributed to C-H bending and the bands at 980 cm^−1^, 971 cm^−1^, and 963 cm^−1^ were associated with a C-O-C vibration in (CH_2_CH_2_O)n. The bands noted at 629 cm^−1^, 624 cm^−1^, and 621 cm^−1^ (Brij 35, Brij 56, and Brij 58P) were considered to correspond with the aliphatic C-H vibration of the CH_2_ groups. A weak band can be noticed at 3855 cm^−1^ (Brij 35), 3835 cm^−1^ (Brij 56), and 3845 cm^−1^ (Brij 58P), and it is supposed that this represents Fe-O bending, in which the direct bond between Fe atoms and Brij 35, Brij 56, and Brij 58P molecules through O atoms and the small band to 616, 609 and 602 cm-1 are attributed at the Fe-surfactant complex, and the circumstance establishes that there was chemisorption performed on the OLC 45 substrate. Comparing Figure 9A–F, it can be supposed that the surfactants Brij 35, Brij 56, and Brij 58P were adsorbed onto the OLC 45 sample substrate. This fact was confirmed using the Langmuir adsorption isotherm examinations.

### 3.5. Surface Investigation using SEM (Scanning Electron Microscopy)

**Figure 10:**
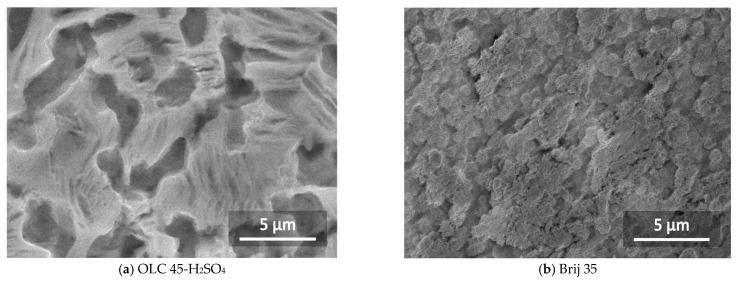

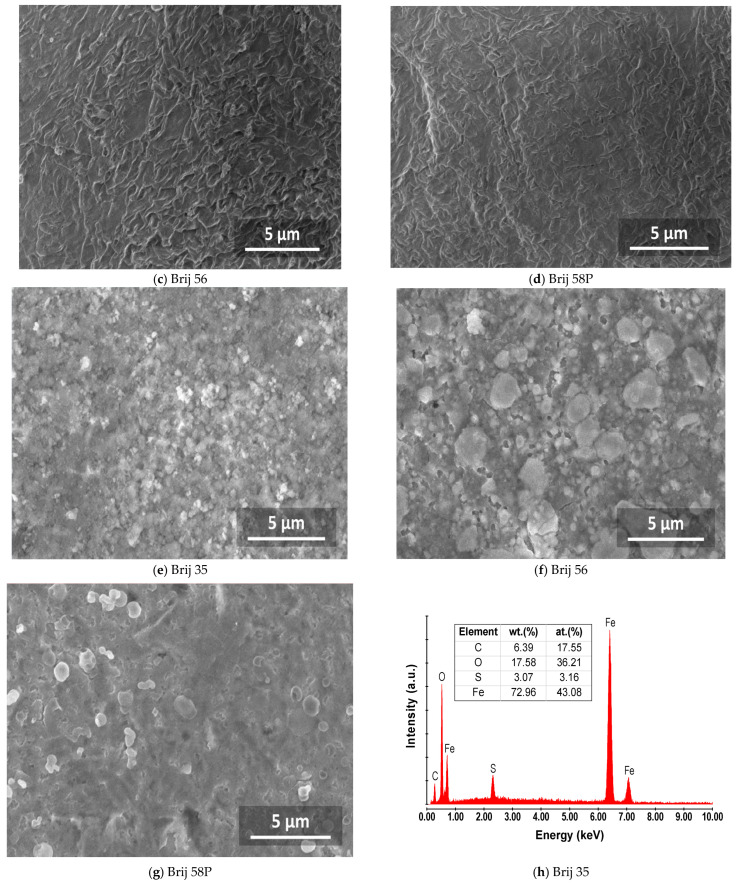

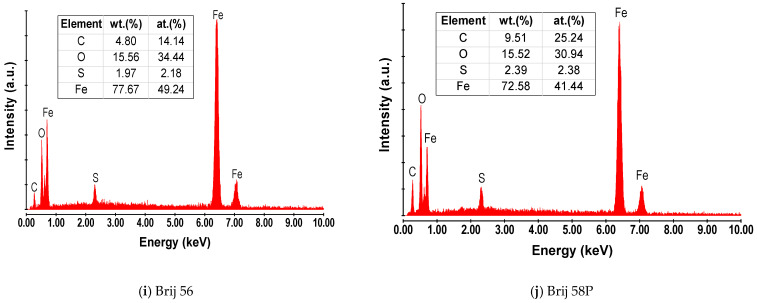
SEM images of OLC 45 electrode in 0.5 M H_2_SO_4_ (**a**) and with 800 ppm (**b**) Brij 35 (**c**), Brij 56 (**d**), Brij 58P; and after immersion time of 120 h (**e**) Brij 35 (**f**), Brij 56 (**g**), Brij 58P; and (**h**–**j**) EDS plots of the Brij/OLC 45.

SEM micrographs taken of the immersion of the OLC 45 surface specimen in the 0.5 M H_2_SO_4_ environment in the absence and presence of the 800 ppm Brij 35, Brij 56, and Brij 58P surfactants are displayed in Figure 10. Figure 10a represents the SEM micrographs of the substrate of OLC 45 sample with immersion in 0.5 M H_2_SO_4_ media, indicating that the OLC 45 substrate was severely deteriorated in the absence of the surfactant. It can be remarked in Figure 10b–e that with the Brij (Brij 35, Brij 56, and Brij 58P at 800 ppm) surfactants, the OLC 45 substrate zone presented a superior and improved morphological substrate compared to that of the uninhibited OLC 45 substrate, showing an appreciable ability of the surfactants to protect against corrosion. An adsorbed film is presented on the OLC 45 substrate to produce a diminution in contact (touch) with the OLC 45 specimen and the aggressive environment, which is liable for the corrosion inhibition [11,40,46,47,48].

The existence of protective films over the OLC 45 substrate was identified in the component peaks of C, O, Fe, and S in the EDS spectra (Figure 10h–j) [40,46,47,48]. These results were in accordance with the FTIR determinations of the inhibited OLC 45. With immersion times between 0 and 192 h in a 0.5 M H_2_SO_4_ solution, an obvious amendment in the morphology of the protective film substrate was noticed according to the electrochemical data. This occurrence is shown in Figure 10g–i, which divulge the diffusion of corrosive ions SO_4_^2−^ into the protective film.

## 4. Conclusions

Three nonionic Brij surfactants with anticorrosion attributes were examined in this work. The Brij surfactants (Brij 35, Brij 56, and Brij 58P) showed good protection performances for OLC 45 substrate in 0.5 M H_2_SO_4_, whereas the corrosion current was diminished and the efficacy raised by increasing the surfactant dosage.

The electrochemical procedure specified that anodic metal oxidation and cathodic hydrogen release mechanisms are considerably hindered by these Brij surfactants, and the EIS determinations disclosed that the charge transfer resistance increased with the surfactant dosage, which shows the accomplishment of a shielding (defensive) layer over the OLC 45 substrate through the adsorption of surfactant molecules which increased the protective activity. Consequently, the inhibited substrate could successfully protect the OLC 45 from corrosion for a long time.

The adsorption of the surfactants Brij 35, Brij 56, and Brij 58P was explored on the OLC 45 substrate following the Langmuir isotherm and it exhibited chemisorption and physisorption.

The value of the Gibbs free energy of adsorption was negative, which indicated the spontaneousness of the adsorption activity.

An exploration and consideration of the substrate with FT-IR, SEM, and EDS facilitated the realization of an inhibitory film on the metal substrate and showed the defense operation of the Brij surfactant on the OLC 45 area in a 0.5 M H_2_SO_4_ electrolyte.

The corrosion protection efficiency of Brij 35 was very close to that of Brij 58P, and both were superior to Brij 56. Consequently, Brij 35 had the highest performance for low concentrations whereas Brij 58P was superior at higher concentrations, and was followed by Brij 56. The large hydrophobic group of Brij surfactants constitutes a good barrier between the OLC 45 substrate and an aggressive environment.

In conclusion, the Brij surfactants prevented the offensive of the corrosive factor-H_2_SO_4_- on the OLC 45 substrate and provided protection to the substrate, as they exhibited a good adsorption performance, implying a considerable anticorrosive defense ability of the inhibitory films.

## Figures and Tables

**Table 1:** Kinetic parameters of OLC 45 in 0.5 M H_2_SO_4_ at certain concentrations of Brij 35 at 25 °C.

Concentration (ppm)	i_corr_ (mAcm^−2^)	R_p_ Ωcm^−2^	R_mpy_	P_mm/year_	K_g_ g/m^2^h	E (%)	−E_corr_ (mV)	b_a_ (mVdec^−1^)	−b_c_ (mVdec^−1^)	θ
0	0.721	21	337	8.54	7.66	-	482	82	84	-
20	0.057	105	27.55	0.69	0.49	92	400	40	63	0.92
50	0.056	106	26.13	0.66	0.59	92	402	39	67	0.92
100	0.049	125	22.86	0.58	0.52	93	435	40	71	0.93
300	0.042	130	19.6	0.49	0.44	94	400	41	60	0.94
500	0.041	110	19.13	0.48	0.43	94	403	43	53	0.94
800	0.032	196	14.93	0.38	0.34	95	417	47	72	0.96
1000	0.027	220	12.6	0.32	0.28	97	414	44	55	0.97

**Table 2:** Kinetic parameters of OLC 45 in 0.5 M H_2_SO_4_ at certain concentrations of Brij 56 at 25 °C.

Concentration (ppm)	i_corr_ (mAcm^−2^)	R_p_ Ωcm^−2^	R_mpy_	P_mm/year_	K_g_ g/m^2^h	E (%)	−E_corr_ (mV)	b_a_ (mVdec^−1^)	−b_c_ (mVdec^−1^)	θ
0	0.721	21	337	8.54	7.66	-	482	82	84	-
20	0.096	164	44.8	1.14	1.01	86	406	51	89	0.86
50	0.0955	156	44.56	1.13	1.01	86	402	65	86	0.86
100	0.047	140	21.93	0.55	0.49	93	405	40	51	0.93
300	0.037	188	17.26	0.43	0.39	94	422	60	68	0.94
500	0.031	160	14.46	0.37	0.33	96	403	43	50	0.96
800	0.023	208	10.73	0.27	0.24	97	402	43	50	0.97
1000	0.029	303	13.53	0.34	0.30	96	429	41	75	0.96

**Table 3:** Kinetic parameters of OLC 45 in 0.5 M H_2_SO_4_ at certain concentrations of Brij 58P at 25 °C.

Concentration (ppm)	i_corr_ (mAcm^−2^)	R_p_ Ωcm^−2^	R_mpy_	P_mm/year_	K_g_ g/m^2^h	E (%)	−E_corr_ (mV)	b_a_ (mVdec^−1^)	−b_c_ (mVdec^−1^)	θ
0	0.721	21	337	8.54	7.66	-	482	82	84	-
20	0.091	111	51.33	1.30	1.17	85	460	74	85	0.87
50	0.087	118	40.6	1.03	0.92	86	460	41	55	0.88
100	0.057	140	26.6	0.67	0.60	92	402	43	64	0.92
300	0.031	216	14.46	0.37	0.33	95	420	44	66	0.95
500	0.025	195	11.66	0.29	0.26	96	398	42	51	0.96
800	0.022	207	10.26	0.26	0.23	97	387	47	60	0.97
1000	0.028	352	13.06	0.33	0.29	97	460	65	64	0.97

**Table 4:** EIS characteristics of OLC 45 with Brij35 in 0.5 M H_2_SO_4_ at 25 °C.

Concentration (ppm)	R_S_ (ohm.cm^2^)	Q − Yo S·s^−n^·cm^−2^	Q − n	R_ct_ (ohm × cm^2^)	χ^2^	E%
0	0.78	0.0065	0.78	18	4.764 × 10^−3^	-
20	1.96	0.001495	0.85	137	2.347 × 10^−3^	87
50	2.41	0.001022	0.91	139	8.138 × 10^−4^	88
100	1.97	0.000181	0.91	178	2.456 × 10^−3^	90
300	2.52	0.0008204	0.92	179	1.154 × 10^−3^	90
500	2.2	0.0008469	0.92	187	1.649 × 10^−3^	91
800	2.04	0.000133	0.92	199	7.275 × 10^−4^	92
1000	1.18	0.0009295	0.93	251	2.897 × 10^−3^	93

**Table 5:** EIS characteristics of OLC 45 with Brij 56 in 0.5 M H_2_SO_4_ at 25 °C.

Concentration (ppm)	R_S_ (ohm.cm^2^)	Q − Yo S·s^−n^·cm^−2^	Q − n	R_ct_ (ohm × cm^2^)	χ^2^	E%
0	0.78	0.0065	0.78	18	4.764 ×10^−3^	-
20	2.81	0.00537	0.89	138	2.274 × 10^−3^	87
50	2.34	0.00142	0.93	152	2.299 × 10^−3^	88
100	3.11	0.00167	0.89	163	1.890 × 10^−3^	89
300	2.12	0.001062	0.92	169	9.114 × 10^−4^	89
500	2.76	0.000661	0.90	231	8.436 × 10^−4^	92
800	3.98	0.000591	0.88	233	7.430 × 10^−4^	92
1000	1.68	0.000176	0.92	241	2.988 × 10^−3^	93

**Table 6:** EIS characteristics of OLC 45 with Brij 58P in 0.5 M H_2_SO_4_ at 25 °C.

Concentration (ppm)	R_S_ (ohm.cm^2^)	Q − Yo S·s^−n^·cm^−2^	Q − n	R_ct_ (ohm × cm^2^)	χ^2^	E%
0	0.78	0.0065	0.78	18	4.764 × 10^−3^	-
20	2.15	0.0003376	0.83	117	8.791 × 10^−4^	85
50	1.93	0.0005022	0.88	121	5.879 × 10^−4^	86
100	2.37	0.0001274	0.85	125	9.0981 × 10^−4^	86
300	3.08	0.000625	0.91	139	7.788 × 10^−4^	87
500	2.11	0.000665	0.90	179	1.117 × 10^−3^	90
800	1.89	0.0002374	0.92	239	2.153 × 10^−3^	92
1000	2.19	0.0001626	0.89	243	5.802 × 10^−4^	93

**Table 7:** The values of E_a_, ΔH°, and ΔS° for three surfactants on OLC 45 in 0.5 M H_2_SO_4_.

Inhibitor	E_a_ (KJ/mol)	ΔH° (KJ/mol)	ΔS° (J/mol K)
Brij 35	69	67	−48
Brij 56	52	48	−95
Brij 58P	73	76	−41
H_2_SO_4_	42	39	−113

**Table 8:** The values of K_ads_ and $\Delta G_{ads}^{\circ }$ for the Brij surfactants on OLC 45 in 0.5 M H_2_SO_4_.

The System	K_ads_, M^−1^	$\Delta G_{ads}^{\circ }$ KJmol^−1^	The Adsorption
Brij 35/OLC 45/H_2_SO_4_	2.19 × 10^5^	−39.7	Chemisorptions and physical adsorption
Brij 56/OLC 45/H_2_SO_4_	6.3 × 10^4^	−36.7	Chemisorptions and physical adsorption
Brij 58P/OLC 45/H_2_SO_4_	2.14 × 10^5^	−39.6	Chemisorptions and physical adsorption

**Table 9:** Comparison of the inhibition efficiency of Brij surfactants with other formerly published corrosion inhibitors (surfactants).

Inhibitor	Substrate	Efficiency	References
2,2’-(1-aminoethane-1,2 diyl)bis(1-(2 aminoethyl)-1-dodecyl-4,5-dihydro-1H-imidazol-1ium)dichloride	X-65 steel	88%	[9]
Tween 60	OL 37	96%	[15]
Tween 80	OL 37	95%	[15]
Span 60	OL 37	92%	[15]
Span 80	OL 37	96%	[15]
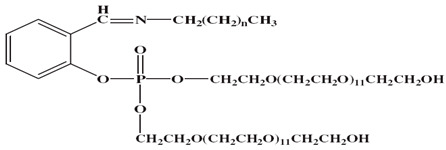	Carbon steel	94%	[22]
Triton X100	Brass/OL 37	67%/95%	[23]
Triton A 20	Brass/OL 37	83%/94%	[23]
DBBD	Mild steel	98%	[42]
QBBD	Mild steel	98.5%	[42]
NaPACD	OLC 45	97%	[47]
NaPADH	OLC 45	95%	[47]
NaPADD	OLC 45	96%	[47]
Tween 85	CRS	92%	[49]
Brij 30	Mild steel	88%	[59]
Brij 35	Mild steel	89%	[60]
Brij 35	OLC 45	97%	present work
Brij 56	OLC 45	96%	present work
Brij 58P	OLC 45	97%	present work

## Data Availability

Data are contained within the article.
